# The structure of a pectin-active family 1 polysaccharide lyase from the marine bacterium *Pseudoalteromonas fuliginea*

**DOI:** 10.1107/S2053230X2400596X

**Published:** 2024-06-27

**Authors:** Joanne K. Hobbs, Alisdair B. Boraston

**Affiliations:** a Department of Biochemistry and Microbiology University of Victoria PO Box 1700 STN CSC Victoria BCV8W 2Y2 Canada; University of York, United Kingdom

**Keywords:** polysaccharide lyases, pectinases, marine bacteria, *Pseudoalteromonas*, pectinate

## Abstract

*Pseudoalteromonas fuliginea* sp. PS47 is a recently identified marine bacterium that has extensive enzymatic machinery to metabolize polysaccharides, including a locus that targets pectin-like substrates. This locus contains a gene (locus tag EU509_03255) that encodes a pectin-degrading lyase, called *Pf*PL1, that belongs to polysaccharide lyase family 1 (PL1). The 2.2 Å resolution X-ray crystal structure of *Pf*PL1 reveals the compact parallel β-helix fold of the PL1 family. The back side of the core parallel β-helix opposite to the active site is a meandering set of five α-helices joined by lengthy loops. A comparison of the active site with those of other PL1 enzymes suggests a catalytic mechanism that is independent of metal ions, such as Ca^2+^, but that substrate recognition may require metal ions. Overall, this work provides the first structural insight into a pectinase of marine origin and the first structure of a PL1 enzyme in subfamily 2.

## Abstract 2

The structure of *Pf*PL1, the first structure of a polysaccharide lyase family 1 subfamily 2 enzyme, reveals a parallel β-helix fold. Structural comparisons reveal insights into substrate recognition and key elements in the catalytic machinery.

## 1. Introduction

*Pseudoalteromonas* is a globally distributed genus that is typically associated with marine environments, including the colonization of macroalgal surfaces (Martin *et al.*, 2014 ▸; Bowman *et al.*, 1997 ▸). *P. fuliginea* sp. PS47 is a recently identified bacterium that was isolated from the surface of macroalgae harvested from the marine environment of the Pacific Northwest. This bacterium has the ability to process carrageenan, agarose and pectin-like polysaccharides (Pluvinage *et al.*, 2020 ▸; Hettle *et al.*, 2019 ▸; Hobbs *et al.*, 2019 ▸).

Pectin is a complex polysaccharide found in the cell walls of plants, particularly in fruits such as apples, citrus fruits and berries. It is primarily composed of chains of α-1,4-linked d-galacturonic acid residues (homogalacturonan), although some forms also contain l-rhamnose in the backbone (rhamnogalacturonan). The complexity of pectins is increased by varying degrees of branching, acetylation and/or methyl­esterification, such as in rhamnogalacturonan II (RGII), which is considered to be one of the most complex polysaccharides in nature. Pectin is not common to macroalgae, but aquatic plants do have pectin-like polysaccharides. Zosterin, which is an apiogalacturonan, is a pectin-like molecule from the aquatic seagrasses in the *Zostera* genus. It has a homogalacturonan backbone substituted with O3-linked β-d-apiofuranose residues, or short chains thereof. Acetyl and methylester modifications have also been detected (Lv *et al.*, 2015 ▸). This ‘marine pectin’ is, therefore, a possible substrate for bacteria with the appropriate enzymatic machinery.

Polysaccharide lyase family 1, PL1, is a family of primarily polygalacturonan/pectin lyases that catalyse depolymerization through a β-elimination mechanism, resulting in the formation of a double bond between the C4 and C5 atoms of the sugar residue at the nonreducing end of the polysaccharide chain (Zheng *et al.*, 2021 ▸). This family is further stratified into 13 subfamilies (Lombard *et al.*, 2010 ▸). *P. fuliginea* sp. PS47 has a pectin-degradation locus that encodes a PL1 referred to as *Pf*PL1 (previously known as *Ps*PL1), in addition to 19 other proteins comprising the machinery for pectin depolymerization, transport and assimilation (Hobbs *et al.*, 2019 ▸). *Pf*PL1 is crucial to initiate pectin depolymerization and has demonstrated activity on apiogalacturonan (Hobbs *et al.*, 2019 ▸). This enzyme belongs to the largest PL1 subfamily, subfamily 2 (denoted PL1_2), which is one of only two subfamilies with characterized members but no available structures (subfamily 8 is the other). Four additional members of PL1_2 have been functionally characterized as pectin lyases: three from the homogalacturonan polysaccharide-utilization locus of *Bacteroides thetaiotaomicron* (BT_4115, BT_4116 and BT_4119) and one from *Dickeya dadantii* (PelZ) (Luis *et al.*, 2018 ▸; Kita *et al.*, 1996 ▸). Here, we report the structure of *Pf*PL1, which is the first structure of a PL1_2 protein. A comparison of the *Pf*PL1 structure with the structures of PL1 enzymes from other subfamilies reveals similarities in substrate recognition but potential differences in the catalytic machinery.

## 2. Materials and methods

### 2.1. Macromolecule production

The gene encoding *Pf*PL1 from *P. fuliginea* sp. PS47 (locus tag EU509_03255; GenBank KAA1163790.1), lacking the predicted secretion signal peptide (amino-acid boundaries 29–464), was cloned as described previously into pET-28a as an in-frame fusion with an N-terminal His_6_ tag to generate pET-28a-PfPL1 (Hobbs *et al.*, 2019 ▸). Protein production and purification were performed as outlined previously with the addition of a final size-exclusion chromatography step using a Sephacryl S-200 column (GE Healthcare) pre-equilibrated with 20 m*M* Tris–HCl pH 8.0, 150 m*M* sodium chloride (Hobbs *et al.*, 2019 ▸). The purified protein was concentrated to ∼8–11 mg ml^−1^ using a stirred-cell ultrafiltration device with a 10 000 Da molecular-weight cutoff membrane (Millipore) for crystallization. Macromolecule-production information is summarized in Table 1 ▸.

### 2.2. Crystallization

Initial crystallization trials were carried out using the commercially available screens Index (Hampton Research) and MSCG1 (Anatrace) by the sitting-drop vapour-diffusion method at 291 K with a 1:1 ratio of protein (8 mg ml^−1^ in 20 m*M* Tris–HCl pH 8.0, 150 m*M* sodium chloride) and crystallization solutions (0.5 µl each). An initial hit was optimized using the hanging-drop vapour-diffusion method and protein at 11 mg ml^−1^, resulting in improved crystals using 20%(*w*/*v*) PEG 3350, 0.1 *M* sodium malonate pH 7.0. Crystallization information is summarized in Table 2 ▸.

### 2.3. Data collection and processing

Only a single suitable crystal was obtained and this was cryoprotected in crystallization solution supplemented with 20%(*v*/*v*) 2-methyl-2,4-pentanediol prior to mounting directly in a nitrogen stream at 100 K. Diffraction data were collected on an instrument comprising a PILATUS 200K 2D detector coupled to a MicroMax-007 HF X-ray generator with a VariMax-HF ArcSec Confocal Optical System and an Oxford Cryostream 800. The data were integrated, scaled and merged using *HKL*-2000. Data-collection and processing statistics are summarized in Table 3 ▸.

### 2.4. Structure solution and refinement

The crystal structure of *Pf*PL1 was solved by molecular replacement with *Phaser* (McCoy *et al.*, 2007 ▸) using a search model of *Pf*PL1 generated with *AlphaFold* (Jumper *et al.*, 2021 ▸; Varadi *et al.*, 2022 ▸). The model was corrected by manual building with *Coot* (Emsley *et al.*, 2010 ▸) and refinement with *phenix.refine* (Liebschner *et al.*, 2019 ▸). The addition of water molecules was performed with Find Waters in *Coot* and manually checked after refinement. Refinement procedures were monitored by flagging 5% of all observations as ‘free’ (Brünger, 1992 ▸). Model validation was performed with *MolProbity* (Chen *et al.*, 2010 ▸). All model statistics are shown in Table 4 ▸.

## 3. Results and discussion

### 3.1. Overall structure of *Pf*PL1

*Pf*PL1 crystallized in space group *P*2_1_2_1_2_1_, with unit-cell parameters *a *= 48.50, *b* = 58.13, *c* = 149.08 Å. *MATTHEWS_COEF* analysis (Kantardjieff & Rupp, 2003 ▸) indicated the presence of a single protein molecule in the asymmetric unit, with a Matthews coefficient of 2.16 Å^3^ Da^−1^ and a solvent content of 43.14%. Analysis with *PISA* (Krissinel & Henrick, 2007 ▸) indicated the absence of noncrystallographic dimers that would be stable in solution. Residues 25–458 of the recombinant protein could be modelled and refined with no gaps in the protein backbone.

**Figure 1:**
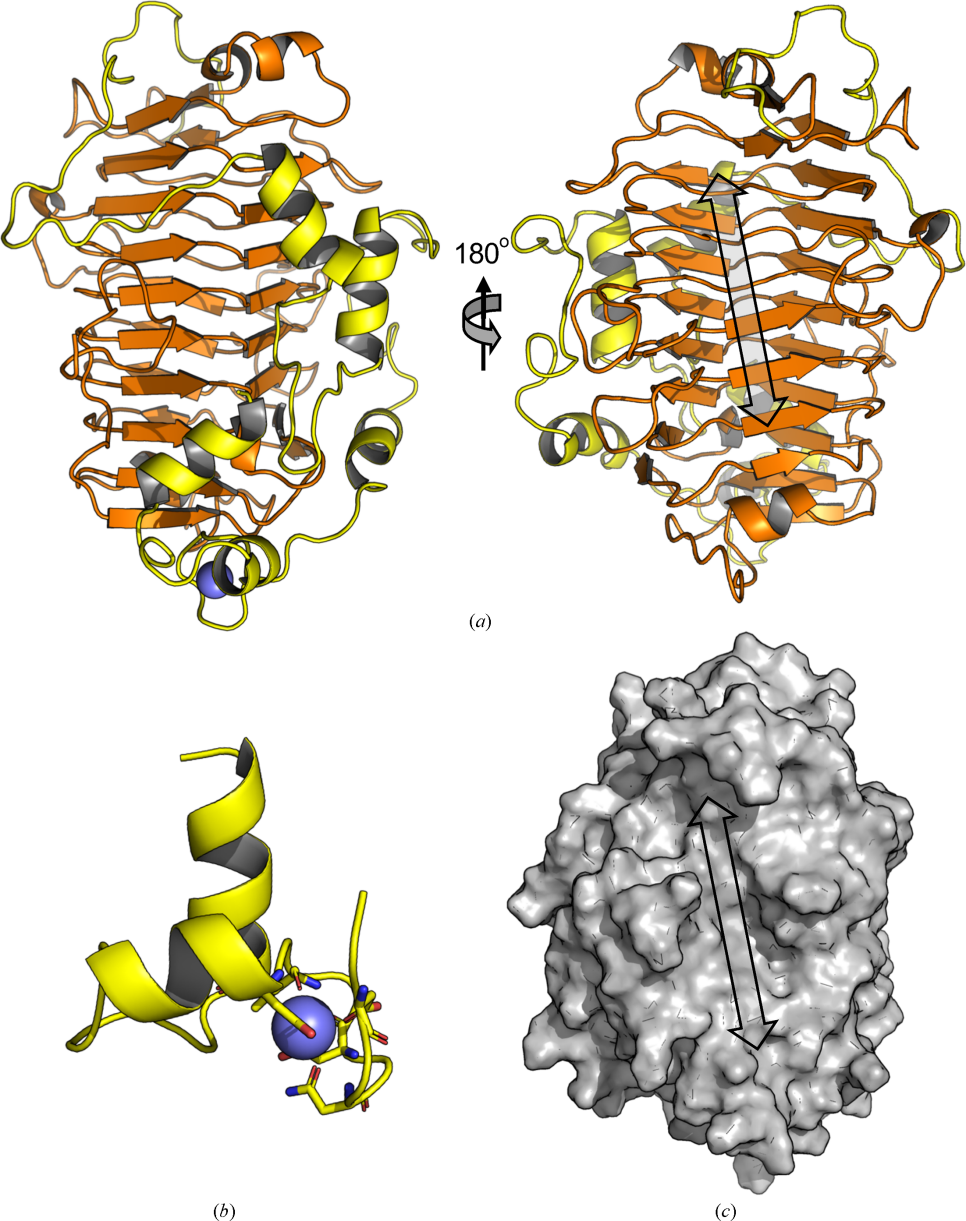
Overall structure of *Pf*PL1. (*a*) Cartoon representation of *Pf*PL1 shown from both sides. The core parallel β-helix is shown in orange and the C-terminal meandering adornment is shown in yellow. (*b*) An enlargement of the pseudo-EF-hand motif found at the C-terminus of *Pf*PL1. Residues involved in coordinating the Ca^2+^ ion are shown as sticks. (*c*) Surface representation of *Pf*PL1 shown as a solvent-accessible surface. The arrows in (*a*) and (*c*) represent the location of the catalytic groove.

The structure of *Pf*PL1 (PDB entry 9buj) comprises at its core the parallel β-helix common to family 1 polysaccharide lyases (Fig. 1 ▸*a*). This compact fold of ∼300 amino acids at the N-terminus is adorned by an ∼125-amino-acid C-terminal region made up of α-helices and large loops that meander across the parallel β-helix surface (Fig. 1 ▸). The C-terminal region terminates in a structural motif resembling an EF-hand; however, in this case the calcium (Ca^2+^) ion is bound on what would be the back of the thumb rather than in the palm at the junction of the thumb and forefinger (Fig. 1 ▸*b*). The *B* factor of the Ca^2+^ ion is comparable to the average *B* factor of the protein, indicating that it is relatively well ordered and thus may represent a structural component that pins together these secondary-structure elements at the C-terminus.

**Figure 2:**
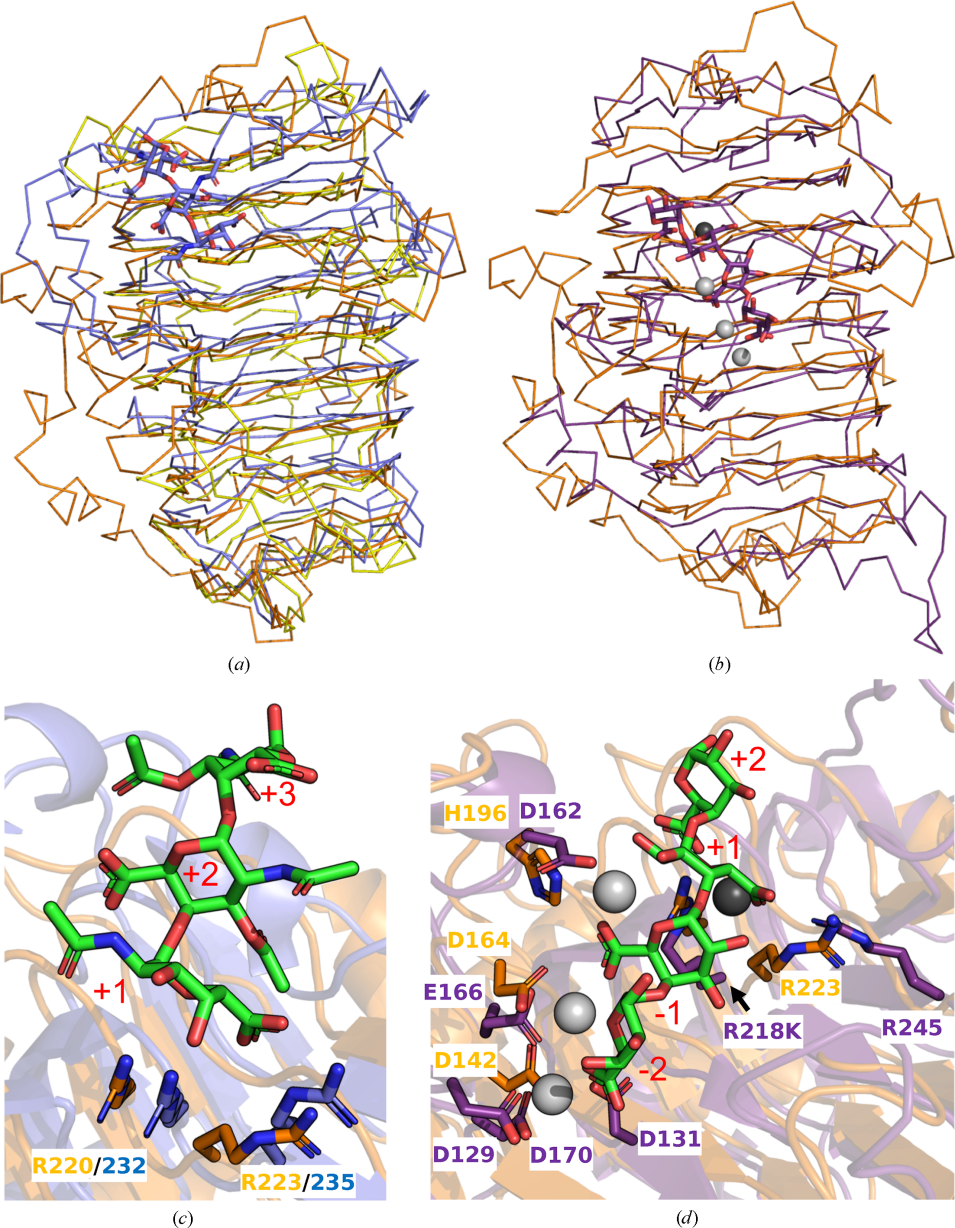
Comparison of *Pf*PL1 with other PL1 enzymes reveals insight into its active site. (*a*) Overlay of *Pf*PL1 (orange), VexL (PDB entry 6fi2, blue) and Jun a 1 (PDB entry 1pxz, yellow) shown as C^α^ ribbons. (*b*) Overlay of *Pf*PL1 (orange) and PelC (PDB entry 1ewe, purple) shown as C^α^ ribbons. (*c*) Active site of *Pf*PL1 (orange) compared with that of VexL (PDB entry 6fi2, blue). (*d*) Active site of *Pf*PL1 (orange) compared with that of PelC (PDB entry 1ewe, purple). Ca^2+^ ions involved in substrate binding in PelC are shown as light grey spheres. The proposed catalytic Ca^2+^ ion involved in the catalytic mechanism of PelC is shown as a dark grey sphere.

A structural similarity search using the *DALI* server returned members of PL1 as the top hits. The two most similar structures were VexL from *Achromobacter denitrificans* [PDB entry 6fi2; *Z*-score and root-mean-square deviation (r.m.s.d.) of 31.7 and 2.4 Å, respectively, over 284 matched C^α^ residues; 21% amino-acid sequence identity] and Jun a 1, the major pollen allergen from *Juniperus ashei* (PDB entry 1pxz; *Z*-score and r.m.s.d. of 31.3 and 2.8 Å, respectively, over 290 matched C^α^ residues; 23% amino-acid sequence identity) (Fig. 2 ▸*a*; Liston *et al.*, 2018 ▸; Czerwinski *et al.*, 2005 ▸). VexL belongs to subfamily 13 of PL1, while Jun a 1 belongs to subfamily 1 (Fig. 2 ▸*a*). Amongst the next four most similar structures was PelC from *Dickeya chrysanthemi* EC16 (PDB entry 1air; *Z*-score and r.m.s.d. of 24.8 and 3.0 Å, respectively, over 269 matched C^α^ residues; 17% amino-acid sequence identity; Fig. 2 ▸*b*), which belongs to subfamily 6 and a mutant of which has notably been captured in complex with intact substrate (PDB entry 2ewe; Scavetta *et al.*, 1999 ▸). The catalytic groove of *Pf*PL1 was identified by comparison to these other PL1 structures and runs along the face of one β-sheet (Fig. 1 ▸*a*, right). The groove is relatively shallow (Fig. 1 ▸*c*), perhaps to assist in the accommodation of decorated pectins, such as the apiogalacturonan found in the marine environment, on which we have shown *Pf*PL1 to be active (Hobbs *et al.*, 2019 ▸).

### 3.2. Structure of the catalytic groove

To provide some insight into the potential interactions of *Pf*PL1 with substrate, we compared its structure with the complexed structures of VexL (PDB entry 6fi2) and PelC (PDB entry 1ewe) in some detail. The VexL structure is a product complex with a trisaccharide of α-1,4-linked *N*-acetylgalacturonic acid occupying subsites +1 to +3 (Liston *et al.*, 2018 ▸). An overlap of this structure with *Pf*PL1 clearly reveals conservation of the catalytic arginine residues, Arg220 and Arg235, in *Pf*PL1 (Fig. 2 ▸*c*). Based on their interaction with VexL, the side chain of Arg220 would abstract the proton from C5 and the side chain of Arg235 would help to neutralize the negative charge on the C6 carboxylate. Some pectin lyases rely on calcium ions for their catalytic mechanism; however, VexL is reported to be metal-independent. The similarity in the arrangement of the catalytic machineries of the two proteins suggests that *Pf*PL1 also does not rely on metals for its catalytic mechanism. This is supported by the overlay of *Pf*PL1 with the PelC complex, an enzyme whose catalytic mechanism is proposed to rely on Ca^2+^. The complex of PelC, obtained by mutating the catalytic arginine to lysine, has α-1,4-tetragalacturonic acid spanning subsites −2 to +2 (Fig. 2 ▸*d*; Scavetta *et al.*, 1999 ▸). A catalytic Ca^2+^ ion in PelC is bound in the pocket and is sandwiched between the substrate and the protein. *Pf*PL1 does not seem to possess an analogous pocket (Figs. 1 ▸*c* and 2 ▸*d*). However, in PelC additional Ca^2+^ ions in the minus (−) subsites are involved in bridging the interaction of the substrate with residues in the active site. Some of the side chains involved in coordinating these ‘binding’ Ca^2+^ ions are structurally conserved in *Pf*PL1 (Fig. 2 ▸*d*). Taken together, these comparisons suggest a Ca^2+^-dependent mode of substrate binding for *Pf*PL1 that is similar to PelC but a catalytic mechanism that does not rely on metals.

## 4. Conclusion

The crystal structure of *Pf*PL1 from the marine bacterium *P. fuliginea* sp. PS47 is the first for the largest subfamily of PL1, subfamily 2. The structure reveals relatively simple loops forming the walls of the active site to create a relatively shallow catalytic groove when compared with other PL1 enzymes. The arrangement of the active site suggests that substrate binding relies on metal atoms to bridge the substrate to active-site residues, but that the catalytic mechanism does not require metals.

## Supplementary Material

PDB reference: *Pf*PL1, 9buj

## Figures and Tables

**Table 1:** Macromolecule-production information

Source organism	*Pseudoalteromonas fuliginea* sp. PS47
DNA source	Genomic DNA
Forward primer†	GCCGCGCGGCAGCCAACTCGACTCAAATTTAGCCTTTAAAAATGC
Reverse primer†	GCTCGAATTCGGATCGATTACTCCGTAATCGAATTTATATAAGC
Expression vector	pET-28a
Expression host	*Escherichia coli*
Complete amino-acid sequence of the construct produced‡	**MGSSHHHHHHSSGLVPRGSHMAS**LDSNLAFKNADGYGKYTQGGRDGKIYIVNSLEDNPKNPAKGTLRHALKRKYKRTVVFNISGVIHLKEPIIVKSGFLTIAGQTSPGGITVAGAPVQVSDADHIIIRYMRFRLGTFKLAEDSMSVRNSRDIIIDHCSFSWSVDETASFYNNQRFTLQNSIVAASLNHSIHPKGHHGYGGIWGGNKASFINNVIAHHNSRTPRLNGSRLKPPYDEQFEFVEFSNNIIFNWGSNNVYGSENGRFNLINNIYKPGPASKAIQLVDLWYSPNITKSQAYISGNYFVGDEKITADNRLGVNYRTSKDAKRKNISMDDKRLSRVKLEPINGAVNSATINSTQKTYSTLIKEKNVGANFNANGMFLDNIDTQVLNQVDGSTPINGKGLINSELEMIKSWEEYERQFLGFPDIIDKNKDGINDRWAAKNPTNQHNINAYINSITE

† The vector sequences used for In-Fusion cloning are underlined.

‡ The His_6_-tag sequence is shown in bold.

**Table 2:** Crystallization

Method	Vapour diffusion, hanging drop
Temperature (K)	291
Protein concentration (mg ml^−1^)	11
Buffer composition of protein solution	20 m*M* Tris–HCl pH 8.0, 150 m*M* sodium chloride
Composition of reservoir solution	20% PEG 3350, 0.1 *M* sodium malonate pH 7.0
Volume and ratio of drop	2 µl, 1:1
Volume of reservoir (µl)	500

**Table 3:** Data collection and processing Values in parentheses are for the outer shell.

Diffraction source	Rigaku MicroMax-007 HF rotating anode
Wavelength (Å)	1.5418
Temperature (K)	100
Detector	Dectris PILATUS 200K
Rotation range per image (°)	0.25
Total rotation range (°)	128
Exposure time per image (s)	180
Space group	*P*2_1_2_1_2_1_
*a*, *b*, *c* (Å)	48.50, 58.14, 149.08
α, β, γ (°)	90, 90, 90
Mosaicity (°)	0.77–1.01
Resolution range (Å)	20.00–2.20 (2.24–2.20)
Total No. of reflections	91830
No. of unique reflections	21844
Completeness (%)	97.8 (82.0)
Mutliplicity	4.2 (3.1)
〈*I*/σ(*I*)〉	14.4 (3.2)
*R*_p.i.m._	0.048 (0.168)
*R*_meas_	0.103 (0.300)
CC_1/2_	0.984 (0.866)
Overall *B* factor from Wilson plot (Å^2^)	31.6

**Table 4:** Structure solution and refinement Values in parentheses are for the outer shell.

Resolution range (Å)	19.50–2.19 (2.29–2.19)
Completeness (%)	96.0
σ Cutoff	*F* > 0.000σ(*F*)
No. of reflections, working set	21445 (2181)
No. of reflections, test set	1057 (106)
Final *R*_cryst_	0.185 (0.210)
Final *R*_free_	0.252 (0.268)
Cruickshank DPI	0.24
No. of non-H atoms	
Protein	3417
Ligand (Ca^2+^)	1
Water	202
Total	3620
R.m.s. deviations	
Bond lengths (Å)	0.009
Angles (°)	1.075
Average *B* factors (Å^2^)	
Protein	32.45
Ligand (Ca^2+^)	33.17
Water	36.29
Ramachandran plot	
Favoured regions (%)	93.3
Allowed (%)	6.7
Outliers (%)	0.00
PDB code	9buj
